# Modality and Perceptual-Motor Experience Influence the Detection of Temporal Deviations in Tap Dance Sequences

**DOI:** 10.3389/fpsyg.2017.01340

**Published:** 2017-08-02

**Authors:** Mauro Murgia, Valter Prpic, Jenny O, Penny McCullagh, Ilaria Santoro, Alessandra Galmonte, Tiziano Agostini

**Affiliations:** ^1^Department of Life Sciences, University of Trieste Trieste, Italy; ^2^Division of Psychology, De Montfort University Leicester, United Kingdom; ^3^Department of Kinesiology, California State University, East Bay, Hayward CA, United States; ^4^Department of Medical, Surgical and Health Sciences, University of Trieste Trieste, Italy

**Keywords:** temporal deviation, rhythm, detection, auditory perception, visual perception, biological motion, temporal information, tap dance

## Abstract

Accurate temporal information processing is critically important in many motor activities within disciplines such as dance, music, and sport. However, it is still unclear how temporal information related to biological motion is processed by expert and non-expert performers. It is well-known that the auditory modality dominates the visual modality in processing temporal information of simple stimuli, and that experts outperform non-experts in biological motion perception. In the present study, we combined these two areas of research; we investigated how experts and non-experts detected temporal deviations in tap dance sequences, in the auditory modality compared to the visual modality. We found that temporal deviations were better detected in the auditory modality compared to the visual modality, and by experts compared to non-experts. However, *post hoc* analyses indicated that these effects were mainly due to performances obtained by experts in the auditory modality. The results suggest that the experience advantage is not equally distributed across the modalities, and that tap dance experience enhances the effectiveness of the auditory modality but not the visual modality when processing temporal information. The present results and their potential implications are discussed in both temporal information processing and biological motion perception frameworks.

## Introduction

Many activities in which we engage are based on temporal information processing. For instance, playing musical instruments, dancing, and performing aesthetic sports are all activities in which accurate temporal information processing is fundamental. Musicians, dancers, and athletes, alike, are exposed to rhythmic stimuli, daily, and are often required to produce movements that are temporally related to what they perceive. For instance, pianists play in synchronization with other musicians and ballet dancers and synchronized swimmers perform in concert with their colleagues/teammates to music. In such activities, successful execution of complex human movement is contingent upon accurate perception of both auditory and visual rhythms that are often associated with the movements of others (i.e., biological movement perception). Overwhelmingly, however, previous research examining temporal information processing has examined relatively simple and lab-based stimuli (e.g., auditory beats, flashing lights, rotating bars; e.g., Grahn, 2012). For this reason in the current study, we examined the role of auditory and visual modalities in temporal information processing and the role of perceptual-motor experience in biological movement perception of tap dance sequences performed by a skilled human model.

### The Role of Auditory and Visual Modalities in Temporal Information Processing

Humans do not use a dedicated sense (i.e., modality) for collecting temporal information from the environment; rather, temporal properties of events can be perceived through different modalities (i.e., visual, auditory, tactile, kinesthetic). However, the modalities involved in temporal information processing are not equally effective, and differences between auditory and visual modalities are well documented in the research literature. Results have generally indicated superiority of the auditory over the visual modality when participants engage in temporal information processing tasks common in lab-based research designs (Repp and Penel, 2002, 2004; Grondin and McAuley, 2009; Grondin, 2010; Stauffer et al., 2012).

To better understand the literature concerning temporal information processing it is important to clarify two important attributes of temporal sequences: rhythm and tempo. A rhythm can be defined as a *pattern* of time intervals demarcated by sensory and/or motor events (Chen et al., 2008), while tempo is the *speed* at which a sequence of these events progresses. In other words, tempo represents how fast a given rhythm is presented. Typically, tempo is described in terms of beats per minute (bpm), while rhythm is described as the ratio of the time intervals (e.g., 1:1:2:1). For instance, a sequence having an inter-onset interval of 250, 250, 500, 250 ms can be reproduced with a faster tempo, while maintaining constant rhythmic structure (e.g., 125, 125, 250, 125 ms).

The processing of rhythm has been widely studied by researchers in both auditory and visual modalities. For instance, Glenberg et al. (1989) exposed their participants to rhythms produced by sequences of short and long stimuli, either in the auditory or in the visual modality, and asked them to reproduce the temporal sequences. They found that the reproduction of rhythms in the auditory modality condition was superior to that of the visual modality condition. Similarly, an auditory modality advantage was observed by Collier and Logan (2000) in a same-different perceptual task (i.e., identifying whether the current rhythm is the same as, or different than, the target rhythm). More recently, the greater sensitivity of the auditory modality for rhythm perception was further confirmed by Stauffer et al. (2012), who asked participants to detect temporal deviations from regular sequences, while Barakat et al. (2015) found that visual rhythm perception is enhanced to a greater degree when participants receive auditory—rather than visual—training.

It is noteworthy that the auditory advantage for rhythm perception seems to exist across different tasks. For instance, researchers have asked participants to compare two rhythmic sequences using a same-different paradigm (e.g., Collier and Logan, 2000; Barakat et al., 2015), while others have asked participants to detect a temporal deviation from regular intervals and to report whether each sequence was perceived as “regular” or “irregular” (e.g., Rammsayer et al., 2012; Stauffer et al., 2012). The fact the auditory advantage has been found by using different paradigms suggests that this evidence is not bound to a specific task but reflects a more general mechanism.

Overall, the literature indicates that the auditory modality dominates the visual modality for rhythm perception. However, the visual stimuli typically used in the majority of studies (e.g., flashing lights) might be not representative of real world contexts, and the use of moving stimuli might strengthen visual rhythm perception research. In this regard, we are not aware of studies documenting the detection of temporal deviations by using moving visual stimuli, nevertheless other studies on rhythm perception highlighted the potential of this kind of stimuli (e.g., Grahn, 2012). Clearer evidence on the role of moving visual stimuli were found in studies using other timing tasks (i.e., sensory-motor synchronization), in which participants had better synchronization performances compared to flashing lights when using rotating bars, moving fingers and bouncing balls (Hove et al., 2010; Iversen et al., 2015). Given that only few studies have investigated the role of moving stimuli in rhythm processing and obtained promising results, it seems important to further analyze moving stimuli, particularly using perceptual tasks.

### The Role of Perceptual-Motor Experience in Biological Motion Perception

Since the pioneering studies of Johansson (1973, 1976), several aspects of biological movement perception have been investigated, allowing researchers to demonstrate that relatively few visual and/or auditory cues are sufficient to evoke accurate representation of complex movements and for participants to be able to discriminate among various characteristics of perceived events (e.g., type of activity, performer, gender, emotions; Barclay et al., 1978; Repp, 1987; Dittrich et al., 1996; Flach et al., 2004; Loula et al., 2005; Auvray et al., 2011; Hohmann et al., 2011; Sevdalis and Keller, 2011; Murgia et al., 2012; Kennel et al., 2014). Overall, based on previous studies, it seems that a crucial factor for accurate biological movement perception is the perceptual-motor experience of the observer (Abernethy et al., 2001; Repp and Knoblich, 2004; Mann et al., 2007; Hohmann et al., 2011; Tomeo et al., 2013).

The role of perceptual-motor experience is addressed within the Theory of Event Coding (TEC; Hommel et al., 2001). The TEC postulates the existence of a common coding system in which perceptual and motor events are in a state of continuous mutual influence. Within this framework, Hommel et al. (2001) note that observers process and interpret stimuli more accurately when the stimuli are more consistent with their own perceptual-motor repertoire (i.e., their previous perceptual-motor experiences). This idea is in line with evidence suggesting an active role of the motor system in biological movement perception (Calvo-Merino et al., 2006; Schütz-Bosbach and Prinz, 2007; Murgia et al., 2016).

In recent years, researchers have further investigated biological movement perception, trying to better understand the peculiarities of the ecological sounds associated with complex human movements (for a review, see Pizzera and Hohmann, 2015). For instance, it has been demonstrated that relying on solely auditory information expert participants can detect opponents’ movement intentions in basketball (Camponogara et al., 2017) and can discriminate shot power in ball sports (Sors et al., 2017). Moreover, Woods et al. (2014) found that listening to sport sounds differentially activates the motor areas of the brain in expert compared to novice athletes, while Murgia et al. (2016) found that ecological sounds of breathing affected breath duration more so than artificial sounds with the exact same temporal structure. Thus, it seems that perceptual-motor experience plays an important role in the processing of ecological sounds and visual stimuli related to complex human movements.

### The Present Study

Our literature review has highlighted that there is a general advantage of the auditory modality over the visual modality for temporal information processing, although in some cases the use of moving stimuli instead of flashing lights can reduce this auditory advantage. On the other hand, researchers of biological movement perception suggest that the perceptual-motor experience of participants is a moderating factor of accurate perception in both auditory and visual modalities. The combination of temporal information processing and biological movement perception has rarely been addressed by researchers, nevertheless, it represents an interesting research challenge which might reveal how athletes, dancers, and musicians process temporal information related to complex human movements.

In the present study, we combined these two areas of research to examine biological movement perception in a temporal information processing framework. In particular, we investigated how temporal deviations in auditory and visual tap dance sequences performed by a human model could be detected by both skilled participants (i.e., tap dancers) and unskilled participants (i.e., university students with no prior tap dance experience). It is noteworthy that the use of these stimuli allowed us to simultaneously investigate the role of modality and that of perceptual-motor experience related with the stimuli. In sum, our aims were: (1) to determine whether the auditory advantage for rhythm perception occurs for biological motion stimuli, and (2) to investigate the potential role of perceptual-motor experience in rhythm perception with biological motion stimuli.

## Materials and Methods

### Participants

Eighteen tap dancers (4 males, 14 females; *M*_age_ = 24.28 years; *SD* = 3.85) and 18 university students (5 males, 13 females; *M*_age_ = 23.67; *SD* = 2.00) participated in the current study. Dancers had at least 3 years of experience in tap dance (*M* = 8.11; *SD* = 3.38) and trained between 2 and 7 h per week (*M* = 4.17; *SD* = 1.89). Students had no prior formal experience in tap dancing or other forms of dance, music, or aesthetic sports. All participants declared they had no hearing limitations and they had normal or corrected-to-normal vision. Participants did not receive money for their participation. Informed consent was obtained for each participant prior to data collection.

### Materials and Apparatus

A tap dance instructor volunteered to serve as the skilled model for the creation of the auditory and visual stimuli. A music player connected with Sennheiser HD515 headphones (total harmonic distortion <0.2%) was used to provide the instructor with a metronome during the recording phase. A Stage Line ECM-925P microphone and a Sony HDRCX105E camera were used to record the stimuli. The microphone was connected with a laptop computer, and the input signal was recorded using Goldwave (*v*5.58). The same software was used to edit and to analyze the sounds. The movies were edited with Microsoft Movie Maker (*v*2.6). An ASUS X52J 15.6′′ LCD display laptop computer, connected with Sennheiser HD515 headphones, was used to administer the stimuli.

### Stimuli Generation

**FIGURE 1:**
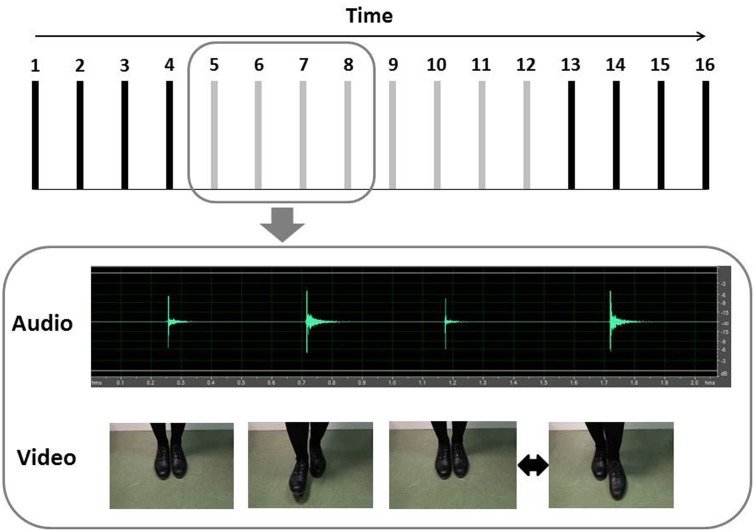
Illustration of the stimuli. The structure of tap dance sequences is shown above, with each bar representing a step. In the RE trials, there was no temporal deviation. In the IR trials, the black bars represent the steps that were always regular and the gray bars represent the steps in which the temporal deviation (early or late step) could occur. In the lower half of the figure, we present a sample IR trial, in the auditory and visual modalities. In this exemplar, the temporal deviation occurred at the eighth step.

A database of auditory and visual stimuli was generated by recording the instructor while performing a sequence of 16 tap steps, called “paddle,” in synchronization with an isochronous sequence of beats (i.e., a metronome set at 132 bpm). The database included a set of “regular” and “irregular” tap dance sequences. To create the regular sequences, the instructor performed 100 trials in synchronization with the metronome. To create the irregular sequences, the instructor performed another 100 trials in synchronization with the metronome, but intentionally committed one slight error in each trial. The error consisted of a temporal deviation of one single step (performed slightly before or after the metronome beat) somewhere in the middle of the sequence, in a self-selected random position between the 5th and the 12th step (see **Figure 1**).

For each trial, we recorded both the movement of the feet (visual information) and the sound they produced (auditory information). The videos were recorded from a frontal perspective, and focused only on the instructor’s feet. The decision to record the stimuli from this perspective was based on the rationale that this is the same visual perspective adopted by individuals practicing tap dance when they train in front of a mirror. The auditory stimuli (i.e., the sound of the tap dancing) were recorded by placing the microphone near the instructor’s feet, at a distance of approximately 40 cm. All trials were performed in a quiet room with only the skilled model and lead researcher present.

To validate the differential tap dance performance trials (regular and irregular), two other tap dance instructors independently rated all visual and auditory trials from 1 (poor) to 5 (excellent). In regards to the “regular” trials (RE), the instructors evaluated the general quality of the performance, the absence of other possible confounding variables (e.g., variations in accents), and whether they perceived the rhythm was actually regular. In regards to the “irregular” trials (IR), the instructors again evaluated the quality and the regularity of the performance (except for the off-beat step), the absence of confounding variables, and the detectability of the temporal deviation (they were told that the deviation had to be neither too slight nor too evident).

To select the tap dance performance trials to be used for the experiment, we considered only the trials that were scored as “excellent” by both instructors (a score of 5). Next, the temporal difference (in milliseconds) between the metronome beats and each step for each trial was calculated. RE trials were retained if all temporal difference values for a given trial were ≤40 ms. IR trials were retained if they met the same criteria as RE trials except for the off-beat step, whose deviation had to range between 60 and 85 ms. Using these criteria, we randomly selected 10 trials for each category of tap dance sequences (RE and IR). The video and audio portions of each trial were isolated from each other, thus enabling the creation of 20 visual stimuli (10 RE and 10 IR) and 20 auditory stimuli (10 RE and 10 IR).

### Design and Procedure

The protocol was approved by the Institutional Review Board at the University wherein data collection took place. A 2 × 2 mixed design was employed. The independent variables were Modality (i.e., audio vs. video) and Experience (i.e., experts vs. non-experts); the former was within-subjects, the latter between-subjects. We measured how participants detected the temporal deviations, by identifying RE and IR trials.

Participants wore headphones and were seated in front of the computer at a distance of approximately 50 cm from the screen. Participants were asked to determine whether the sequences of tap steps they would listen to/watch were RE or IR. Before beginning the experimental phase, participants performed a practice session comprised of six trials for each modality condition. The practice data were excluded from the analysis. The experimental phase consisted of two blocks of 20 trials (i.e., one block of auditory stimuli and one of visual stimuli; 40 trials, total). For each of the 40 trials, participants were asked to verbally report whether they felt the tap sequence they just heard/saw was RE or IR, prior to moving onto the next trial. The order of stimuli was randomized within each block and the order of the blocks (audio–video or video–audio) was counter-balanced. No feedback about response accuracy was provided during the experiment.

## Results

**FIGURE 2:**
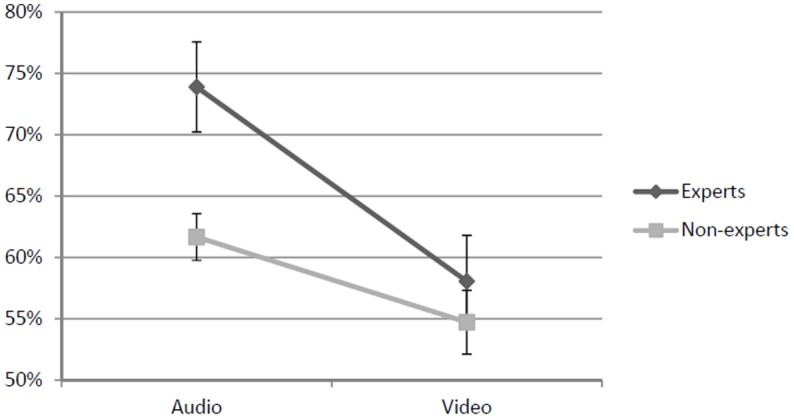
Graphic representation of the results. This graph shows the percentage of accurate responses in the auditory and visual conditions, both for experts and non-experts. Error bars indicate standard errors.

We calculated the percentage of correct responses for each participant, resulting in an average of 73.9% in the auditory condition and 58.1% in visual condition for the experts, and 61.7% in the auditory condition and 54.7% in the visual condition for the non-experts (**Figure 2**). Then, we calculated the *d′* scores for each participants and used these scores as a measure of response accuracy for the statistical tests.

We ran a preliminary set of one-sample *t*-tests which revealed that the response accuracy was above the chance level in all conditions. This was verified for the experts in both auditory [*t*(17) = 5.94; *p* < 0.001; *d* = 1.4] and visual conditions [*t*(17) = 4.16; *p* < 0.001; *d* = 0.98], and for the non-experts in both auditory [*t*(17) = 3.05; *p* < 0.005; *d* = 0.72] and visual conditions [*t*(17) = 1.82; *p* < 0.05; *d* = 0.43].

In order to investigate whether Modality and Experience affected the response accuracy, we used a 2 × 2 mixed ANOVA. The results revealed significant main effects for both Modality [*F*(1, 34) = 17.93; *p* < 0.001; $\eta_{p}^{2}$ = 0.35], and Experience [*F*(1, 34) = 5.89; *p* < 0.05; $\eta_{p}^{2}$ = 0.15], with higher accuracy scores for the auditory modality (compared to the visual modality) and for the experts (compared to the non-experts), respectively, while the interaction approached significance[*F*(1, 34) = 3.29; *p* = 0.078; $\eta_{p}^{2}$ = 0.09]. A set of Bonferroni-adjusted *post hoc* comparisons revealed that accuracy in the auditory modality was higher than in the visual modality in the group of experts (*p* = 0.001), but not in the group of non-experts (*p* = 0.09). Moreover, in the auditory modality the experts were more accurate than non-experts (*p* = 0.02), while in the visual modality no difference was observed (*p* = 0.33).

## Discussion

Previous studies on temporal information processing have revealed a general advantage for the auditory modality over the visual modality (Glenberg et al., 1989; Glenberg and Jona, 1991; Repp and Penel, 2002, 2004; Grondin and McAuley, 2009; Grondin, 2010; Stauffer et al., 2012). Research on biological movement perception have revealed that perceptual-motor experience is a crucial factor for accurate perception of human movement and its attributes (Abernethy et al., 2001; Repp and Knoblich, 2004; Mann et al., 2007; Hohmann et al., 2011; Tomeo et al., 2013). We combined these two areas of research and used biological motion stimuli to examine temporal information processing; in particular, we examined participants’ ability to detect temporal deviations in tap dance sequences performed by a skilled model. We manipulated the modality of presentation and tested two groups of participants with different perceptual-motor experience, and found an advantage for the auditory modality over the visual modality and for experts over non-experts. *Post hoc* analyses suggested that these results were mainly due to the detection abilities exhibited by experts in the auditory modality.

In line with the majority of studies concerning modality differences in rhythm processing (Glenberg et al., 1989; Glenberg and Jona, 1991; Collier and Logan, 2000; Rammsayer et al., 2012; Stauffer et al., 2012), our results indicated superior accuracy of the auditory modality in detecting temporal deviations. However, it is interesting to note that while this finding was quite apparent in the group of experts, it was less evident in the group of the non-experts (i.e., a significant modality difference was not observed in non-experts). The relatively small observed differentiation in performance between the auditory and visual modalities in the group of non-experts might be interpreted in different ways. It might be due to the use of moving visual stimuli or, alternately, it is possible that a floor effect occurred, since the percentage of accuracy was quite low in both auditory and visual modalities (barely above the chance level, in the visual modality). The former interpretation would be consistent with other studies showing that the use of moving visual stimuli can lead to temporal information processing performances close to those observed for auditory stimuli (Hove et al., 2013; Iversen et al., 2015), however, we do not believe that our experimental design was comprehensive enough to draw this particular conclusion (as we were not explicitly testing for differential effects of different stimuli). In our opinion, to specifically examine this, it would be necessary to further study the temporal information processing performances of non-experts using different kinds of stimuli (e.g., flashing lights, auditory signals, moving stimuli, etc.) as well as different levels of temporal deviations to examine whether a floor effect might have affected our results.

Perhaps the most interesting finding in our study concerns the role of perceptual-motor experience. In line with biological movement perception literature (Abernethy et al., 2001; Repp and Knoblich, 2004; Mann et al., 2007; Hohmann et al., 2011; Tomeo et al., 2013), we found better temporal information processing performances among our expert- compared to our non-expert-participants, however, the skill level advantage was not equally distributed across the modalities. Indeed, although the main effect for skill level indicated superior detection of temporal deviations by tap dancers compared to university students, *post hoc* analyses showed that the skill level advantage mainly occurred in the auditory (and not in the visual) modality. These differential modality effects are interesting, and require explanation. We hypothesize that perceptual-motor experience with tap dancing improves the sensitivity to rhythms in an asymmetrical way; the auditory—but not the visual—rhythmic sensitivity is strengthened with practice. Initially, this might seem an improbable or arbitrary explanation, as tap dancers have relatively equal exposure to both auditory and visual rhythmic stimuli (i.e., they constantly listen to their own tap dancing performances *and* watch themselves in a mirror during training). However, we suggest that skilled tap dancers may selectively use the auditory modality to focus on temporal aspects of performance and the visual modality to focus on other tap dance performance aspects which rely more heavily on visual stimuli (e.g., posture, floor position, relative limb placements, etc.). This could explain why the ability to detect temporal deviations of experts was similar to non-experts in the visual modality, while it was noticeably superior to that of non-experts in the auditory modality.

A possible interpretation of the present results within the framework of TEC (Hommel et al., 2001) is that the temporal information collected in the visual modality condition did not activate an event representation in the common coding system. Consistent with the TEC framework, if the perceived temporal information did activate an event representation, a match with our expert participants’ perceptual-motor repertoire, and subsequently, superior temporal information processing performance, would have been observed of the experts compared to the non-experts. However, our two skill level groups—in the visual modality condition—performed almost equally, suggesting that the perceived visual temporal information was not relevant enough to achieve an adequate match with experts’ perceptual-motor repertoire. As a consequence, the event representation in the common coding system was probably similar for experts and non-experts, leading to analogous temporal information processing performances. Conversely, in the auditory modality, the perceived temporal information did appear to activate an event representation in the common coding system, thus promoting a match with perceptual-motor repertoire in the group of experts. Consequently, the experts—in the auditory modality condition—exhibited better temporal information processing performances compared to non-experts. To better understand the actual contribution of one’s own perceptual-motor repertoire to the detection of temporal deviations (rather than just a superior sensitivity to temporal information acquired with practice), further studies comparing biological and artificial stimuli are necessary.

A novel point of the present work is that we used a set of biological motion stimuli, unlike the majority of previous studies on temporal information processing (Grondin, 2010) and, in particular, on the detection of temporal deviations (Rammsayer et al., 2012; Stauffer et al., 2012). This allowed us to better understand how temporal information is processed in an ecological situation like dancing. Moreover, by adopting this approach, we have been able to examine the role of perceptual-motor experience with stimuli, a crucial aspect in biological motion perception (Abernethy et al., 2001; Repp and Knoblich, 2004; Mann et al., 2007; Hohmann et al., 2011; Tomeo et al., 2013). From a broader perspective, the present study represents one of the first attempts to combine the domains of biological motion perception and temporal information processing. The combination of these two areas of research might have a potential impact in those motor activities in which timing skills are fundamental. In this regard, further research is needed to clarify how humans process temporal information associated with biological motion stimuli in different situations and tasks, and how these outcomes can be used by practitioners in the fields of sport psychology and motor learning and performance.

From an applied perspective, the present findings have important implications regarding the development of perceptual-motor training (Farrow and Abernethy, 2002; Smeeton et al., 2005; Murgia et al., 2014). Indeed, the acquisition of timing skills is fundamental in dance, music, as well as in many sport activities, and perceptual-motor training aimed at enhancing timing skills can be useful for practitioners. Based on our results, when developing perceptual-motor training programs/interventions, practitioners should take into account that experts in a certain discipline might naturally, and more effectively, process movement-related temporal information in the auditory modality rather than visual modality. Deliberate attempts to present timing-related stimuli as auditory stimuli should be made. In this regard, Sors et al. (2015) have anecdotally noted that the majority of existing training aimed at optimizing athletes’ timing skills are based on sounds; the present study provides empirical support for this potential “best practice.”

In the last few years, research pursuit of the role of sounds in interpreting complex human movement has increased markedly (for a recent special issue, see Murgia and Galmonte, 2015), with particular emphasis on the role of ecological sounds associated with movement (Murgia et al., 2015; Pizzera and Hohmann, 2015; Steenson and Rodger, 2015). Such research has demonstrated that a variety of information can be extracted from ecological sounds by experts (Camponogara et al., 2017; Sors et al., 2017). The present study adds to this body of literature, showing that experts appear to more easily detect temporal deviations in tap dance sequences through ecological sounds compared to ecological visual stimuli, as well as providing preliminary evidence suggesting experts may selectively use the auditory sense when engaging in biological movement perception. We would like to note, however, that in our opinion, performance information embedded within ecological sounds remains insufficiently investigated, as well as the potential applications for deliberate use of the auditory modality during temporal perception to enhance timing-related motor performances (O et al., 2015). Future research is required to further investigate the information conveyed though ecological sounds and to develop new strategies to use this information to improve dance, music, and sport performances.

## Ethics Statement

This study was carried out in accordance with the recommendations of Research Ethics Committee of the University of Trieste with written informed consent from all subjects. All subjects gave written informed consent in accordance with the Declaration of Helsinki. The protocol was approved by the Research Ethics Committee of the University of Trieste.

## Author Contributions

MM, VP, JO, PM, IS, AG, and TA designed the study; MM and TA prepared the stimuli; MM, PM, and IS collected the data; MM and AG performed the statistical analyses; MM, VP, and JO wrote the manuscript; MM, VP, JO, PM, IS, AG, and TA revised the manuscript.

## Conflict of Interest Statement

The authors declare that the research was conducted in the absence of any commercial or financial relationships that could be construed as a potential conflict of interest.
